# Anesthetic management of a patient with musculocontractural Ehlers-Danlos syndrome undergoing scoliosis surgery

**DOI:** 10.1186/s40981-020-00352-5

**Published:** 2020-06-11

**Authors:** Ryo Wakabayashi, Satoshi Tanaka, Keiko Tsuchiyama, Katsumi Yamamoto, Yuki Maruyama, Kaori Numata, Mikito Kawamata

**Affiliations:** Department of Anesthesiology and Resuscitology, Shinshu University School of Medicine, 3-1-1 Asahi, Matsumoto, Nagano, 390-8621 Japan

**Keywords:** Massive bleeding, Musculocontractural Ehlers-Danlos syndrome, Viscoelastic hemostatic assay monitoring

## Abstract

**Background:**

Musculocontractural Ehlers-Danlos syndrome is a new and rare subtype of Ehlers-Danlos syndrome in which anesthetic considerations for airway and respiratory management, prevention of skin injuries and joint dislocations, and hemostatic management for severe perioperative bleeding are required.

**Case presentation:**

A 19-year-old woman with musculocontractural Ehlers-Danlos syndrome was scheduled to undergo posterior spinal fusion from the 4th thoracic to the 4th lumbar vertebrae under general anesthesia. Her trachea was easily intubated with a videolaryngoscope despite a small mouth and micrognathia. Pressure-controlled ventilation with limited peak inspiratory pressure was performed for prevention of pneumothorax. Skin damage and joint luxation were prevented by using a low rebounding mattress, terpolymer-based barrier film, and careful patient positioning. Blood transfusion was effectively performed on the basis of point-of-care viscoelastic hemostatic assay monitoring. She had an uneventful postoperative course without any complications.

**Conclusions:**

We safely managed a patient with musculocontractural Ehlers-Danlos syndrome undergoing scoliosis surgery.

## Background

Musculocontractural Ehlers-Danlos syndrome (mcEDS) is a new subtype of EDS that is caused by carbohydrate sulfotransferase 14/dermatan 4-O-sulfotransferase-1 deficiency [1, 2]. mcEDS is an extremely rare subtype of EDS; only 31 patients from 21 families have been reported [1]. mcEDS is clinically characterized by multiple congenital malformations including a small mouth and micro-retrognathia and by progressive multisystem fragility-related complications including pneumothorax or pneumohemothorax, recurrent joint dislocations, and large subcutaneous hematoma [1, 2]. These characteristics are not always present in other subtypes of EDS [2]. The characteristics indicate the need for anesthetic considerations for airway and respiratory management, prevention of skin damage and joint luxation, and hemostatic management for severe perioperative bleeding. However, there has been no report of anesthetic management in a patient with mcEDS. Here, we report safe management of a patient with mcEDS undergoing scoliosis surgery.

## Case presentation

We obtained written informed consent for this case report from the patient and her mother. The patient was a 19-year-old woman (height, 161 cm; weight, 57 kg) with mcEDS. At the age of 1 year, she underwent surgical correction of low anorectal malformation and congenital clubfeet. She recurrently developed skin injuries and limb dislocations. At the age of 11 years, a large subcutaneous hematoma of the skull due to minor trauma occurred, and blood transfusion was required. She was genetically diagnosed with mcEDS at the age of 15 years. Her scoliosis gradually progressed, and she was referred to our hospital for spinal surgery.

**Fig. 1:**
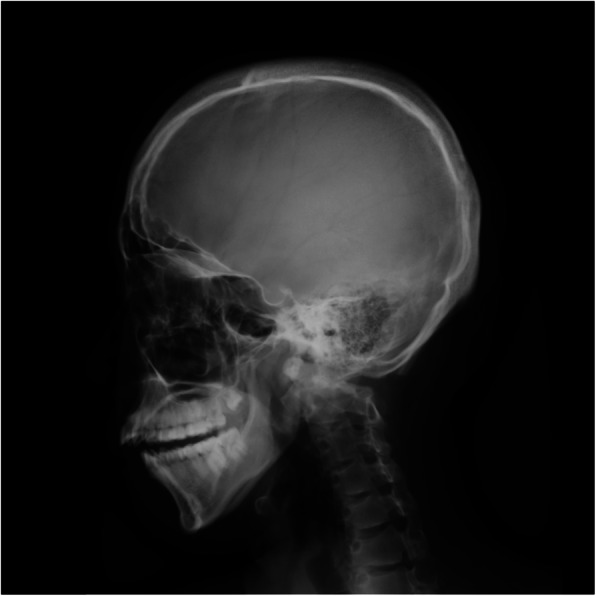
A lateral X-ray image of the head showing micrognathia

Preoperative physical examination revealed a small mouth and micrognathia with a Mallampati score of class III. Skin hyperextensibility and generalized joint laxity were also evident. Preoperative standard laboratory testing indicated normal blood coagulability (Table 1). A lateral X-ray image of the head showed micrognathia (Fig. 1). Computed tomography of the spine revealed right scoliosis from the 3rd to 12th thoracic vertebrae and left scoliosis from the 1st to 5th lumbar vertebrae. A respiratory function test showed restrictive ventilatory impairment with a forced vital capacity of 1800 ml (53% predicted). Other preoperative test results, including results of electrocardiography and echocardiography, were within normal limits. Ten units of fresh frozen plasma (FFP), an equal amount of red blood cells (RBCs), and intraoperative RBC salvage were prepared for surgery.

**Table 1:** Results of standard laboratory testing before and after surgery

	PT-INR (0.85–1.15)	APTT (s) (23.0–38.0)	Fibrinogen (mg/dl) (180–350)	Platelet count (/μl) (158,000–348,000)	Hemoglobin (g/dl) (13.7–16.8)
Before surgery	0.97	28.6	297	359,000	13.4
After surgery	0.99	24.9	243	181,000	12.0

Reference ranges in parentheses are based on the values used in our hospital

*PT-INR* Prothrombin-time international normalized ratio, *APTT* Activated partial-thromboplastin time

For anticipated bleeding during extensive scoliosis surgery, we prepared the following management plans: (1) monitoring of coagulation parameters by using a sonoclot analyzer (Sonoclot coagulation and platelet function analyzer SCP2, Sienco, Inc., Morrison, CO, USA) with a gbACT+ kit at every 500 ml of bleeding and of hemoglobin level by arterial blood gas analysis; (2) FFP transfusion if the clot rate decreased below the normal range; (3) RBC transfusion at a hemoglobin level below 8.0 g/dl; and (4) platelet concentrate transfusion if the platelet function fell below the reference range with a platelet count by standard laboratory testing of less than 50,000/μl. Since difficult intubation was suspected, we prepared a McGRATH™ MAC videolaryngoscope (Medtronic, Dublin, Ireland). To reduce the risk of pneumothorax and pneumohemothorax, we planned to use pressure-controlled ventilation with limited peak inspiratory pressure. A low rebounding mattress and a terpolymer-based barrier film (Cavilon™ no-sting barrier film; 3M, St. Paul, MN, USA) were prepared for skin protection.

In the operating room, she was given intranasal administration of 300 μg 1-desamino-8-D-arginine vasopressin (DDAVP) before induction of general anesthesia. General anesthesia was induced with propofol target-controlled infusion (TCI) set at 2.5 μg/ml and 0.2 μg/kg/min remifentanil. Bag-mask ventilation was easily conducted. After 40 mg rocuronium had been intravenously administered, the trachea was intubated by using a McGRATH™ MAC videolaryngoscope. The Cormack-Lehane grade was 1, and intubation using a 6.5-mm ID tracheal tube was successful in the first attempt. The patient’s lungs were mechanically ventilated by pressure-controlled ventilation with a peak inspiratory pressure of 15–18 cmH_2_O, respiratory rate of 10–12 breaths/min, inspiratory to expiratory ratio of 1:2, and positive end-expiratory pressure of 5 cmH_2_O. Anesthesia was maintained with propofol TCI set at 3.0 μg/ml, 0.1–0.5 μg/kg/min remifentanil, and intermittent bolus of fentanyl (total of 600 μg). A double-lumen central venous catheter was inserted via the right internal jugular vein. Intraoperative monitoring using pulse oximetry, electrocardiogram, noninvasive blood pressure in the left wrist (due to hyperalgesia in response to pressure in the upper arms), invasive right radial artery blood pressure, central venous pressure, end-tidal carbon dioxide pressure, processed electroencephalogram, and rectal temperature was carried out. Position change from supine to prone was safely completed without development of skin damage or joint luxation.

Before skin incision, 17.5 mg/kg tranexamic acid was intravenously administered. The results of clot rate, activated clotting time, and platelet function determined by sonoclot coagulation analysis and hemoglobin level measured by arterial blood gas analysis are summarized in Table 2. At 1000 ml of blood loss, the clot rate and hemoglobin level were below the pre-defined triggers, and we started to transfuse FFP and RBCs. The clot rate, activated clotting time, and hemoglobin level gradually recovered as shown in Table 2. Although the platelet function was below the reference range at 2000 and 2500 ml of blood loss, platelet concentrate transfusion was not performed because laboratory investigations at that time showed a normal platelet count of 136,000/μl, and surgical site bleeding was controllable. Immediately before the end of surgery, 17.5 mg/kg tranexamic acid was intravenously administered again. None of the sonoclot signatures during surgery indicated hyperfibrinolysis.

**Table 2:** Results of point-of-care sonoclot coagulation analysis and arterial blood gas analysis during surgery

Blood loss (ml)	Clot rate (9.0–35.0)	Activated clotting time (s) (100–155)	Platelet function (> 1.5)	Hemoglobin (g/dl) (13.7–16.8)
0	19.6	151	3.3	11.2
500	18.0	157	4.2	9.9
1000	5.3	278	2.9	6.2
1500	8.4	220	3.2	7.2
2000	9.2	197	1.4	8.9
2500	12.9	161	1.3	9.1

Reference ranges for clot rate, activated clotting time, and platelet function shown in parentheses are based on the manufacturer’s report, and the reference range in parentheses for hemoglobin is based on values used in our hospital

The surgery for posterior spinal fusion from the 4th thoracic to the 4th lumbar vertebrae was completed without adverse events. The total operation time was 386 min, and total anesthesia time was 591 min. Ten units of FFP, an equal amount of allogenic RBCs, and 300 ml of autologous RBCs were transfused during surgery. The total amount of fluid infusion was 3250 ml; total blood loss was 2600 ml, and total urine volume was 320 ml. Prone-to-supine position change was carefully performed, and skin injuries and joint dislocations were not evident. The patient was uneventfully extubated in the operating room and transferred to an intensive care unit. A postoperative chest X-ray did not indicate pneumothorax or pneumohemothorax. Postoperative analgesia was effectively provided by intravenous infusion of 0.5 μg/kg/h fentanyl. Standard laboratory testing after surgery indicated normal blood coagulability (Table 1). Amounts of postoperative blood loss were 230 ml within 6 h and 380 ml within 12 h. Additional blood transfusion was not required in the postoperative period. She had an uneventful postoperative course without complications, and she was discharged on postoperative day 37.

## Discussion

Patients with mcEDS are especially prone to the development of spinal deformities [1, 2]. They are at an increased risk of bleeding and other complications during spinal surgery as a result of their connective tissue disorder [3, 4], and posterior instrumented infusion for scoliosis is unfavorably associated with substantial blood loss [5]. According to recommendations for acute bleeding in patients with EDS [6], we performed frequent sonoclot coagulation analysis and aggressive transfusion therapy for massive intraoperative bleeding. The sonoclot analyzer with the gbACT+ kit provides a qualitative graphical display of the clotting process including fibrinolysis and also produces quantitative results of the clot rate, activated clotting time, and platelet function within about 15 min [7, 8]. The intervention contributed to prompt detection and effective improvement of coagulopathy during surgery, resulting in maintenance of normal blood coagulability after surgery despite severe intraoperative bleeding of 2600 ml. We administered DDAVP preoperatively in order to facilitate hemostasis. DDAVP increases plasma levels of factor VII and von Willebrand factor [6], and the duration of its effect is 5–8 h [9]. Previous studies showed that DDAVP treatment after trauma prevented the development of large subcutaneous hematomas in patients with mcEDS [10]. We also used tranexamic acid according to European guidelines [11], and hyperfibrinolysis was not observed on sonoclot signatures throughout surgery.

Other concerns included airway and respiratory management. Although a difficult airway was suspected, intubation was successful at the first attempt by using a McGRATH™ MAC videolaryngoscope. Due to the risk of pneumothorax and pneumohemothorax in patients with mcEDS [1, 2], mechanical ventilation in pressure-controlled ventilation mode with limited peak inspiratory pressure was performed [6].

Even minor shear forces can cause severe decollement injuries in EDS patients with skin fragility [6]. Furthermore, there was an increased risk of medical adhesive-related skin injury possibly resulting in severe skin damage [6]. We used a low rebounding mattress to reduce shear forces and external tissue pressure and a terpolymer-based barrier film to prevent medical adhesive-related skin injury. According to a previous report of a brachial plexus injury due to intraoperative shoulder malposition in an EDS patient [12], we carefully performed patient positioning. Consequently, skin injuries, joint dislocations, and nerve injuries did not occur. mcEDS is a rare variant of EDS. Further accumulation of mcEDS cases is required to improve perioperative management.

In conclusion, we safely managed a patient with mcEDS undergoing scoliosis surgery in whom anesthetic considerations for airway and respiratory management, prevention of skin injuries and joint dislocations, and hemostatic management for severe intraoperative bleeding were required.

## Data Availability

Not applicable

## Abbreviations

APTT: Activated partial-thromboplastin time
DDAVP: 1-Desamino-8-D-arginine vasopressin
FFP: Fresh frozen plasma
mcEDS: Musculocontractural Ehlers-Danlos syndrome
PT-INR: Prothrombin-time international normalized ratio
RBC: Red blood cell
TCI: Target-controlled infusion
